# An anti-TNF-α antibody mimetic to treat ocular inflammation

**DOI:** 10.1038/srep36905

**Published:** 2016-11-22

**Authors:** Hanieh Khalili, Richard W. Lee, Peng T. Khaw, Steve Brocchini, Andrew D. Dick, David A. Copland

**Affiliations:** 1 UCL School of Pharmacy, London, UK; 2 National Institute for Health Research (NIHR) Biomedical Research Centre at Moorfields Eye Hospital NHS Foundation Trust and UCL Institute of Ophthalmology, London, UK; 3 University of East London, School of Health, Sport and Bioscience, Water lane, Stratford campus, London, E15 4LZ, UK; 4 School of Clinical Sciences, University of Bristol, Bristol, UK

## Abstract

Infliximab is an antibody that neutralizes TNF-α and is used principally by systemic administration to treat many inflammatory disorders. We prepared the antibody mimetic Fab-PEG-Fab (FpF_infliximab_) for direct intravitreal injection to assess whether such formulations have biological activity and potential utility for ocular use. FpF_infliximab_ was designed to address side effects caused by antibody degradation and the presence of the Fc region. Surface plasmon resonance analysis indicated that infliximab and FpF_infliximab_ maintained binding affinity for both human and murine recombinant TNF-α. No Fc mediated RPE cellular uptake was observed for FpF_infliximab_. Both Infliximab and FpF_infliximab_ suppressed ocular inflammation by reducing the number of CD45+ infiltrate cells in the EAU mice after a single intravitreal injection at the onset of peak disease. These results offer an opportunity to develop and formulate for ocular use, FpF molecules designed for single and potentially multiple targets using bi-specific FpFs.

Uveitis is a CD4^+^ T-cell mediated, non-infectious inflammatory condition in the eye that can result in blindness^1^. Tumor necrosis factor (TNF-α), is secreted by macrophages, T-cells other cell types including neurons and there are increased concentrations of TNF-α and soluble TNF-α receptors in the aqueous humor of non-infectious uveitis patients^2^,^3^. TNF-α plays a pivotal role in inflammatory responses and neutralizing TNF-α delivers a potent regulation of experimental autoimmune uveoretinitis (EAU)^4^.

Therapeutic antibodies that target TNF-α such as infliximab are widely used to treat inflammatory conditions, e.g. rheumatoid arthritis^5^,^6^,^7^. Off-label use of these antibodies suggests they can be used to reduce ocular inflammation caused by uveitis^8^. However, systemic administration of anti-TNFα antibodies does carry risks of severe adverse reactions (e.g. activation of latent infection, hepatotoxicity, lupus) and is contraindicated in some patients^9^. High doses (e.g. 5 mg/kg)^10^ are necessary to achieve therapeutic levels within the eye. Whilst there is now significant evidence describing the clinical efficacy of anti-TNF therapies, in particular when delivered systemically for treatment of uveitis associated with systemic disorders (e.g. Behcet’s disease), there remains an unmet need to exploit the rapid onset of action of antibodies that target TNF-α through intravitreal (IVT) injection. This would permit the administration of higher and reproducible doses directly to the eye, allowing us to treat isolated intraocular inflammation without need for systemic therapies. Unfortunately, the IVT injection of antibodies such as infliximab that target TNF-α have been associated with elevation of inflammatory markers and increased retinotoxicity in uveitis patients^11^,^12^,^13^,^14^,^15^.

Experimental autoimmune uveitis (EAU) is a mouse model that displays a subacute and more clinically fulminant form of ocular inflammation^4^. The use of many animal models to evaluate the efficacy of therapeutic antibodies that are targeted to human proteins is often limited due to decreased affinity for the murine target, but also due to the formation of anti-drug antibodies. In the current study, we demonstrate that infliximab has strong binding affinity to both mouse and human TNF-α, and *in vivo* administration suppresses infiltration of inflammatory immune cells to the retina. The clinical potential of infliximab was validated using the EAU platform to provide the basis for us to then develop and evaluate an antibody mimetic termed Fab-PEG-Fab (FpF), comprising Fab fragments derived from infliximab. The use of FpF_infliximab_ allowed a direct comparison with infliximab. Our results demonstrate that FpF_infliximab_, which lacks the potentially immunogenic Fc region, is not taken up by retinal pigment epithelium (RPE) cells as is infliximab. FpF_infliximab_ maintains robust binding affinity to TNF-α but importantly is also efficacious in terms of suppressing the acute phase of inflammation in the EAU mouse model.

## Results

### Intravitreal administration of Infliximab suppresses EAU

**Figure 1:**
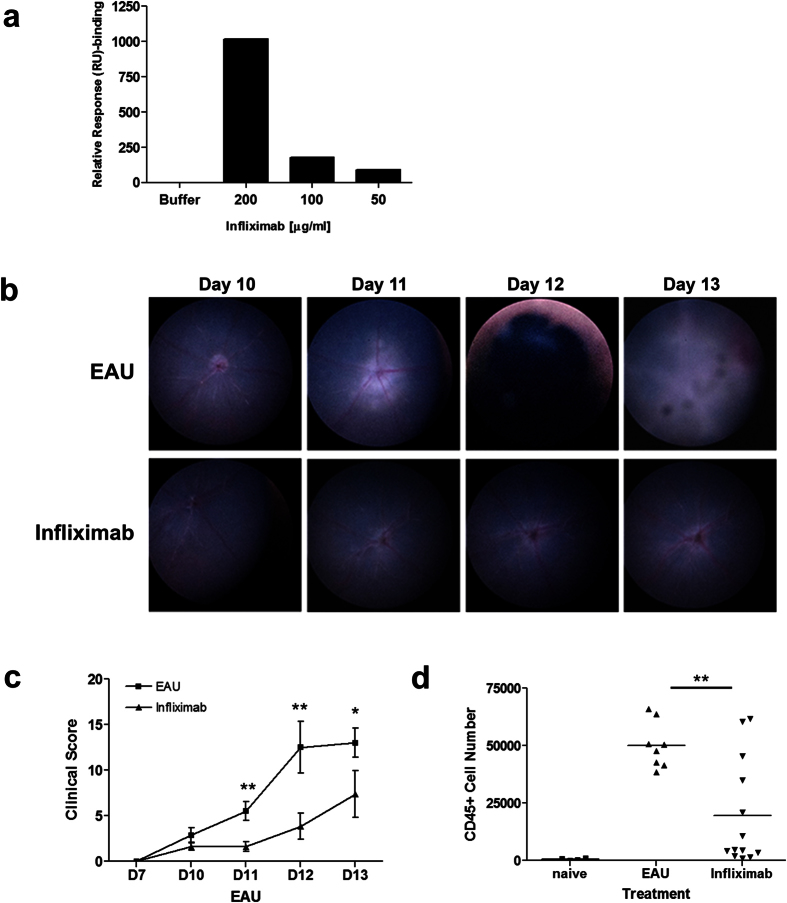
Local administration of infliximab suppresses EAU. (**a**) Graph detailing the Surface Plasmon Resonance (SPR) binding analysis, confirming that infliximab can bind to murine TNF-α using a NTA chip. (**b**–**d**) Mice were immunized for EAU and eyes monitored using TEFI from day 10 onward to select experimental mice displaying clinically evident disease. Groups of mice were injected via intravitreal route with 15 μg infliximab or vehicle control (EAU) on day 10. Eyes were enucleated on day 14, and retinal infiltrate characterized and quantified; (**b**) Representative TEFI images and (**c**) combined total disease scores demonstrating the difference in clinical disease progression between treatment groups. In EAU control eyes typical disease progression with signs of raised optic disc, vasculitis and severe inflammation; In infliximab treated eyes, only raised optic disc and initial signs of vasculitis are evident. (**d**) Graph showing total CD45^+^ infiltrate numbers from individual eyes. *P < 0.05, **P < 0.005, ***P < 0.0005; Data presented as means ± SEM, and representative of two independent experiments.

Infliximab is an IgG_1_, chimeric monoclonal antibody developed to bind to human TNF-α to inhibit its interaction with TNF-receptors. It was not clear from the literature describing systemic use of infliximab in different preclinical models, whether it can bind to mouse TNF-α and therefore enable us to demonstrate efficacy in the EAU model^16^,^17^,^18^. Ocular administration of infliximab has been shown to provide significant retinal and corneal protection in an mouse model of alkali injury^19^, as well as modulating choroidal neovascularization and endotoxin-induced inflammation in rat and rabbit experimental models respectively^20^,^21^. In the current study we therefore sought to confirm the binding of infliximab to both murine (Fig. 1a) and human (Figure S1A) recombinant TNF-α by surface plasmon resonance (SPR) prior to conducting an *in vivo* assessment using the EAU model.

As in previous pre-clinical studies, we then utilised the highly susceptible B10.RIII mouse strain in which the immunising regimen produces consistent moderate disease severity^22^. Using topical endoscopic fundal imaging (TEFI)^23^,^24^, clinical changes which correlate with significant inflammatory cell tissue infiltrate are evident from around day 10 post immunization. During the acute period these are progressive changes that include a swollen optic nerve, peri-vascular infiltrate and vasculitis, vitritis and retinal detachment. These resolve by day 28 leaving a persistent chorioretinal inflammatory infiltrate for months^23^. In the current experiments, TEFI enabled us to screen and select experimental groups of mice that displayed early signs of disease onset, namely raised optic nerve or early vasculitis.

Groups of selected mice were treated on day 10 with a single intravitreal administration of infliximab (15 μg/eye) or control vehicle. The clinical appearance was assessed daily, and the retinal infiltrate examined and enumerated by flow cytometry at 96 hours following dose administration. Clinically at this time point, control treated mice display typical disease features with raised optic disc, vasculitis and choroidal lesions, whereas mice receiving infliximab only exhibited low grade disease (Fig. 1b,c). Flow cytometric analyses of the retinas of the infliximab mice on day 14 display significantly reduced CD45 positive infiltrate (Fig. 1d), reflecting reductions in CD4, CD8, CD11b and Ly6G positive populations (Figure S2A). Overall, there is a 55% reduction of the total CD45^+^ infiltrate (with 68%, 62%, 38% & 64% reductions in CD4, CD8, CD11b and Ly6G populations respectively) in mice receiving infliximab compared to control animals.

### Synthesis of endotoxin-free infliximab mimetic, FpF_Infliximab_

**Figure 2:**
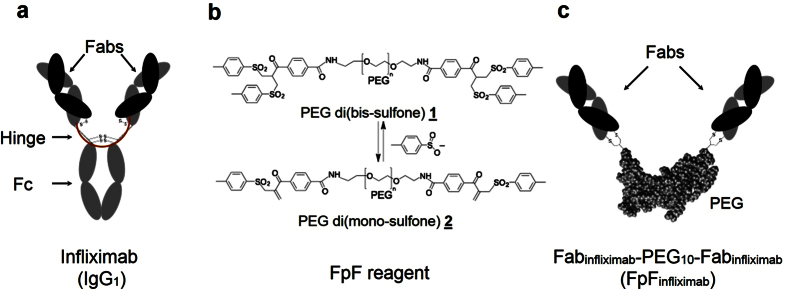
Schematic of Fab-PEG-Fab synthesis from whole IgG. (**a**) Representation of infliximab, an IgG1 with two Fabs that are linked together through the hinge as if each Fab is linked at the end of a linear molecule. Proteolytical digestion of IgG1 yield the Fabs fragment which are then treated with DTT and then linked to each end of PEG di(mono-sulfone) reagent **2** to make FpF, (**b**) PEG di(mono-sulfone) reagent **2** is synthesised from PEG di(bis-sulfone) reagent **1** through elimination of toluene sulfinic acid anion (**c**) The FpF_infliximab_ is synthesized in a way to compare directly to the starting infliximab. This strategy is applicable on different kind of monoclonal IgGs such as human, humanised and chimeric IgGs.

An IgG antibody mimetic called an FpF (Fab-PEG-Fab) was then prepared using the Fabs from infliximab (FpF_infliximab_) and reagent **1** (Fig. 2). The robust suppression of clinical disease evident following intravitreal administration of infliximab, provided the rationale to prepare and evaluate FpF_infliximab_. The Fabs in both an IgG antibody and FpF are at each terminus of a flexible chain that is topologically similar (Fig. 2a,c)^25^. The bis-sulfone moieties in reagent **1** readily undergo elimination to the corresponding mono-sulfone moieties, depicted as PEG-di(mono-sulfone) **2** (Fig. 2b). Conjugation occurs by a sequence of addition-elimination reactions to insert a stable 3-carbon methylene bridge between the two thiols of an accessible disulfide bond in a Fab (Figure S3)^26^. Site-specific conjugation with the two cysteine thiols from the Fab disulfide occurs in the region where the Fab is normally bound to the hinge in an IgG antibody. The thiol ether bonds in the re-bridged disulfide are more stable than the original disulfide bond.

**Figure 3:**
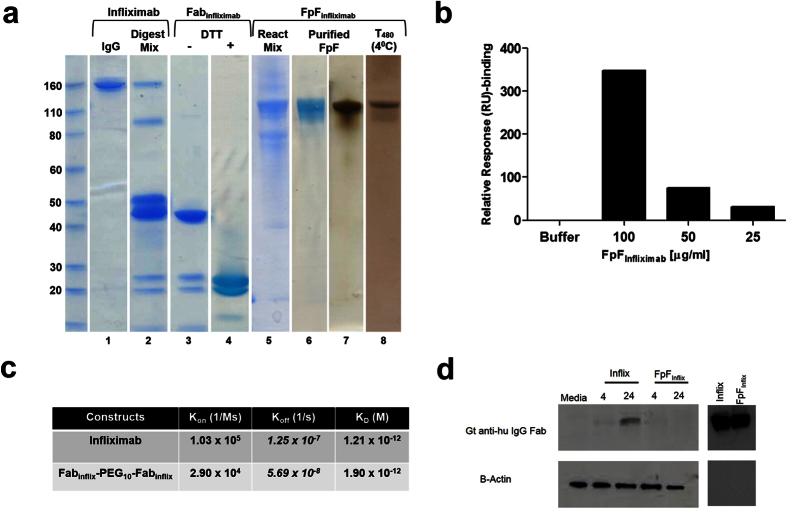
FpF_infliximab_ preparations: assessment of stability, affinity & intracellular uptake. (**a**) Representative Coomassie and silver-stained SDS-PAGE demonstrate the synthesis steps and protein size modifications involved in synthesis of FpF_infliximab_ from whole infliximab (lanes 1–7). FpF_infliximab_ stored at 4 °C for 480 days did not display dissociation from the PEG linker or aggregate formation (lane 8), (**b**) SPR binding analysis demonstrates that FpF_infliximab_ binds murine TNF-α using NTA chip, (**c**) Kinetic affinity (K_D_) and rate constants (k_a_, k_d_) for infliximab and FpF_infliximab_ to recombinant human TNF-α. FpF has similar binding affinity to full IgG, but the dissociation rate constants are 5× slower for FpF_infliximab,_ (**d**) Whole cell lysates prepared from B6RPE-07 cells treated with infliximab or FpF_infliximab_, for 4 & 24 hrs, were probed and the presence of the molecules detected using anti-human IgG Fab. As a negative control, cells were incubated with media alone; and as a positive control preparations of either molecule were applied to the lane.

Using endotoxin free reagents and working under sterile conditions, FpF_infliximab_ was prepared (Fig. 3a). First, infliximab was proteolytically digested with immobilized papain in the presence of cysteine monohydrate (Fig. 3a, lane 2), before elution of the purified Fab_infliximab_ over protein A (lane 3). The Fc fragment appeared at a slightly higher molecular weight on SDS-PAGE than the Fab_infliximab_ due to glycosylation present on the CH2 domain of the Fc fragment. Fab_infliximab_ was then treated with dithiothreitol (DTT) to reduce the accessible disulfide (lane 4).

After removal of DTT by elution over a PD-10 column, the Fab_infliximab_ solution was incubated with PEG di(mono-sulfone) reagent **2** (0.9 equiv.) to obtain FpF_infliximab_ that was then purified by ion-exchange chromatography (lanes 5–6), which was confirmed by silver stain (lane 7). No unconjugated Fab_infliximab_ or the intermediate, (mono-sulfone)-PEG-Fab_infliximab_, were observed. The endotoxin levels of both the purified Fab_infliximab_ after proteolytic digestion of infliximab and the purified FpF_infliximab_ were less than 0.005 EU/mL (EndoZyme assay). There was no heavy-light chain dissociation or aggregation of the FpF_infliximab_ when stored at 4 °C for 480 days (lane 8).

### FpF_infliximab_ displayed slower dissociation rate constant compared to infliximab

SPR analysis demonstrated FpF_infliximab_ displayed concentration dependent binding to human histidine-tag TNFα (Figure S1B). The kinetic rate constants and relative binding affinities (KD) to human TNF-α were calculated using a 1:1 binding model ratio, showing that FpF_infliximab_ and infliximab share similar K_D_ values (Fig. 3c). While the dissociation (k_d_) rate constants was approximately 5 times slower in FpF_infliximab_ compared to infliximab, association rate constant (k_a_) appeared to be faster in infliximab. This is consistent for FpFs^25^ and other bivalent mimetics^27^ that have been evaluated by SPR. The slower dissociation rate constant suggested a tighter interaction between FpFs and TNFα, and potentially improved therapeutic efficacy for FpF_infliximab_. The SPR analysis confirmed FpF_infliximab_ binding to murine TNF-α and warranted an *in vivo* assessment starting at a concentration of 100 μg/mL FpF_infliximab_ (Fig. 3b).

### *In vitro* evaluation of Fc mediated uptake

Antibody uptake into the retinal pigment epithelium (RPE)^28^ can occur via neonatal Fc-receptors expressed in RPE cells^29^,^30^. Uptake of infliximab and FpF_infliximab_ were assessed *in vitro* using a murine B6RPE-07 cell line^31^ in which cell cultures exhibit cobblestone-like morphology similar to primary mouse RPE cultures. The cells also express RPE markers, tight junction and adhesion molecules, and show polarized phenotype when cultured in collagen-coated membrane^31^ and have been used as an *in vitro* RPE model in many recent functional studies^32^,^33^,^34^,^35^. We used RT-qPCR to measure *Fcgrt* mRNA from samples of cultured B6RPE-07 cells, to confirm expression of the transmembrane domain of the neonatal Fc receptor in this cell line (Fig. S4). Confluent RPE cell monolayers were treated separately with 250 μg/ml of each molecule, and cell lysates examined for presence of anti-human IgG Fab fragment. Western blot analysis indicated that intracellular infliximab was detectable in murine B6RPE-07 cells at 4 and more strongly by 24 hrs. In contrast, incubation of FpF_infliximab_ did not result in any detectable Fab protein indicating no intracellular uptake (Fig. 3d).

### FpF_Infliximab_ is efficacious in EAU

**Figure 4:**
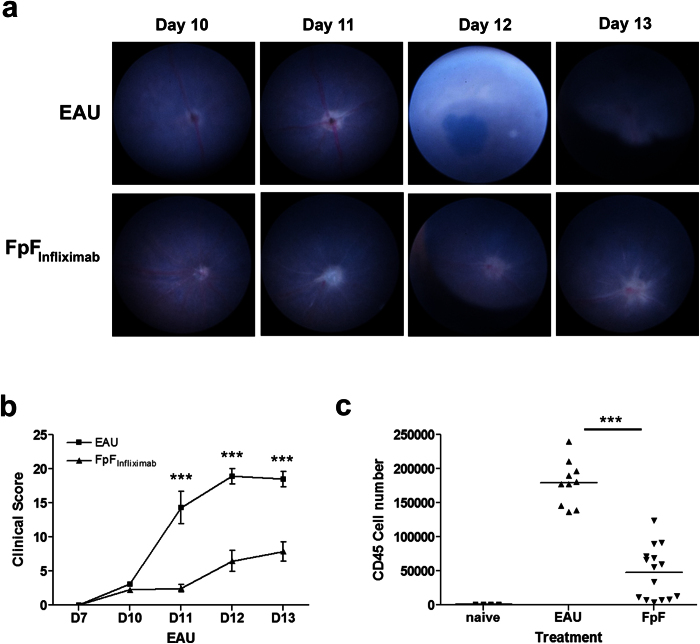
FpF_infliximab_ is efficacious in suppressing disease *in vivo*. Mice were immunized for EAU and eyes monitored using TEFI from day 10 onward to select experimental mice displaying clinically evident disease. Groups of mice received an intravitreal injection of 15 μg FpF_infliximab_ or vehicle control (EAU) on day 10. Eyes were enucleated on day 14, and retinal infiltrate characterized and quantified. Representative TEFI images (**a**) and combined disease scores (**b**) demonstrating the difference in clinical disease progression between treatment groups. Graph showing total CD45^+^ infiltrate numbers from individual eyes (**c**). *P < 0.05, **P < 0.005, ***P < 0.0005; Data presented as means ± SEM, and representative of two independent experiments.

Using the same local administration regimen as described for infliximab, immunized mice displaying the initial signs of clinical disease were selected for treatment groups on day 10, with animals receiving a single intravitreal administration of FpF_infliximab_ (15 μg/eye) or control vehicle. In eyes receiving FpF_infliximab_ only the initial disease changes to the optic disc with some early signs of vasculitis were evident contrasting the expected inflammatory changes and disease severity observed in EAU controls (Fig. 4a,b). At day 14, flow cytometric assessment of retinal infiltrate demonstrates an equally significant reduction of CD45^+^ infiltrate in mice receiving FpF_infliximab_ treatment (Figs 4c and S2B) as compared to infliximab. Overall, there is a 74% reduction of the total CD45+ infiltrate, comprising 69%, 62%, 74% and 82% reductions in CD4, CD8, CD11b and Ly6G populations respectively in mice treated with FpF_infliximab_ compared to control animals.

## Discussion

There is an ever-growing need to develop targeted therapies for use in the eye. Ideally, therapies are needed that are directed to multiple specific targets. We are developing recombinant-conjugation strategies focused on the development of multifunctional and bispecific proteins that are difficult to make entirely by recombinant means alone. For organ specific delivery such as to the eye, there is also a need to ensure new biologically based medicines remain stable throughout the planned duration of action. In this study we started with an anti-TNF-α FpF to determine its ability to achieve biological efficacy in terms of suppressing retinal infiltrate in a clinically relevant, aggressive model of ocular inflammation. TNF-α is involved in the pathogenesis of inflammation, and it has also important roles in progression of undesirable angiogenesis in the eye in the neovascular age-related macular degeneration (AMD) and proliferative vitreoretinopathy diseases^36^,^37^. In this proof-of-concept study we demonstrate for the first time that infliximab suppressed the acute phase of inflammation by reducing the number of CD45+ infiltrate cells in the eyes of the EAU mice. *In vivo*, infliximab and FpF_infliximab_ are equally efficacious as determined by clinical observation while significantly suppressing the infiltration of leukocytes into the retina during peak EAU. This aspect of the study was required before FpF molecules directed to multiple targets can be evaluated.

FpF_infliximab_ synthesis from Fabs obtained by the enzymatic digestion of infliximab allowed the direct comparison of infliximab and FpF_infliximab_. The flexible hydrophilic PEG scaffold replaced the infliximab Fc. The inflammatory reaction after IVT injection of infliximab may be due to Fc related effects or due to antibody degradation or aggregation^38^. An FpF cannot cause localised inflammatory reactions due to the antibody Fc effector functions, which is important when considering treatments for an acute inflammatory condition such as uveitis. Antibody Fc mediated recycling that prolongs the duration of action during systemic use is not critical for ocular pharmacokinetics^39^ and may to some extent remove antibodies from within the eye^30^,^40^,^41^,^42^,^43^.

Since the Fab has an accessible disulfide near the hinge region, each Fab in an FpF is conjugated in essentially the same region that native Fabs are bound to the hinge region in IgG antibodies. The thiol-ether bonds of a re-bridged disulfide are more stable than the starting disulfide. The PEG scaffold is more stable than the single polypeptide chains of the IgG hinge, which are susceptible to cleavage reactions^44^,^45^, and the PEG would reduce the propensity for aggregation^46^,^47^ compared to an IgG. It is thought that the potential for increased stability of FpF_infliximab_ will provide opportunities to make easier-to-use high concentration formulations for direct intravitreal ocular administration than is possible with IgG antibodies. This also raises the possibility of a requirement for less frequent injections compared to the standard antibody.

As TNF-α is a non-covalent trimer in circulation, it is not possible to maintain the trimeric structure of immobilised TNF-α after the regeneration step required for each SPR cycle to remove bound antibody. Since more realistic and comparative results are expected when the ligand, rather than the antibody, is immobilised onto a sensor chip^48^ we used histidine-tagged TNF-α for immobilisation onto a metal affinity chip (nitrilotriacetic acid; NTA). Each regeneration cycle caused removal of the TNF-α, but it was possible to obtain reproducible results by re-immobilising the same amount of TNF-α at the beginning of each SPR sample cycle.

Infliximab and FpF_infliximab_ both displayed binding to mouse TNF-α at high concentration. Binding of both infliximab and FpF_infliximab_ to human TNF-α was also observed, although binding was achieved at lower concentrations than with mouse. Binding of infliximab to mouse TNF-α was important to establish because there have been conflicting observations about infliximab’s binding with mouse TNF-α^16^,^49^. Although the concentrations needed for binding to mouse TNF-α were higher than for human TNF-α, this level of binding was sufficient to evaluate anti-TNF-α molecules in the EAU model. The binding kinetics suggested that FpF_infliximab_ has comparable affinity to infliximab, but slower association and dissociation rates. Slower association rates may be important in the design of new high affinity therapies that display decreased off-target effect. Slower dissociation rates may also be important to ensure that once bound to a ligand, an antibody does not in effect become a slow releasing depot for that ligand. Certainly there are applications were a decreased dissociation rate may be a viable strategy to increase efficacy by increasing the residence time within the target organ or tissue^50^. A potential disadvantage of decreased dissociation rates would be if a target cell surface receptor had agonist function.

The outer blood-retinal barrier, located between the choroid/Bruch’s membrane, exterior to the sub-retinal space and neural retina is formed by the retinal pigment epithelium (RPE), a cell monolayer interconnected by tight junctions. As a selective barrier, the RPE permits intraocular migration of leukocytes as well as a variety of physiological functions including the phagocytosis of shed photoreceptor outer segments and transport of molecules to and from the retina^51^,^52^. Recent data shows that therapeutic anti-VEGF mAbs (bevacizumab and afilbercept) can be transported intracellularly across RPE cells, implicating these cells as important protagonists in the clearance of antibodies from the sub-retinal space into the choroid^28^. Uptake in the RPE is a general feature of Fc-fragment containing molecules such as IgG^28^, via Fc-receptors expressed in RPE cells^29^,^30^. Using *in vitro* RPE cells, no sub-retinal uptake was observed for FpF_infliximab_. The avoidance of RPE uptake and uptake to other cell types such as retinal endothelium^53^ could be important to minimize toxicity for longer acting dosage forms of biologics.

In summary, we have demonstrated that both infliximab and FpF_infliximab_ suppress intraocular inflammation by reducing the number of CD45+ infiltrate cells in the EAU mice model. Clinically, both molecules appeared to be equally effective in modulating the acute inflammatory response that characterizes EAU. FpF_infliximab_ displayed similar binding affinity (K_D_) with slower association and dissociation rates compared to infliximab. There was no Fc mediated RPE cellular uptake of FpF_infliximab_. These characteristics along with a greater potential for FpF stability are being further investigated to determine potential clinical advantages in developing new treatments using the FpF approach that can bind to two different targets.

## Materials and Methods

### Reagents

Complete medium consisted of Dulbecco’s modified Eagle’s medium(DMEM) supplemented with 10% heat inactivated fetal calf serum, 100 U/ml penicillin–streptomycin, 2 mmol/l L-glutamine, 1 mmol/l sodium pyruvate and 5 × 10^−5^ mol/l 2-mercaptoethanol (all from Life Technologies, UK). For FpF_infliximab_ synthesis, preparations of infliximab (Remicade^®^; Janssen Biotech Inc., USA) were used as starting material. Human RBP-3_161–180_ peptide (SGIPYIISYLHPGNTILHVD) was obtained from Severn Biotech (Kidderminster, UK). Peptide purity was determined by HPLC. Peptide preparations were aliquoted and stored at −80 °C.

#### Mice

B10.RIII breeding colony was established in Animal service unit of University of Bristol. All mice were housed under specific pathogen-free conditions with continuously available food and water. The mice were aged between 6 and 8 weeks. Treatment of animals conformed to the Association for Research in Vision and Ophthalmology Statement for the Use of Animals in Ophthalmic and Vision Research. The methods were carried out in accordance with the approved University of Bristol institutional guidelines and all experimental protocols under a Home Office Project Licence 30/3281 were approved by the University of Bristol Ethical Review Group.

### FpF_Infliximab_ synthesis

Using endotoxin (ET) -free glassware and buffers, Fab_infliximab_ fragments were first obtained by enzymatic digestion of infliximab using immobilized papain as described previously^25^. Purified Fab_infliximab_ was isolated from the digestion mixture using ET-free protein A midi kit (Generon, Maidenhead, UK) according to the manufacturer’s instructions. The endotoxin level of purified Fab_inflix_ preparations was less than 0.005 EU/mL as determined using EndoZyme assay.

Under sterile conditions, Fab_infliximab_ (2.4 mg/mL, 6.0 mg in 2.5 mL PBS, pH 7.3) was incubated with dithiothreitol (DTT) (1.0 mg/mL, 2.5 mg) at ambient temperature without shaking for 30 min. DTT was removed by elution over a new PD-10 column, and the protein was buffer exchanged into the conjugation buffer (20 mM sodium phosphate, 10 mM EDTA, pH 7.4). PEG-di(*mono*-sulfone) **2**(0.9 eq, 10 kDa)^25^ was added (1.08 mg) to the reduced Fab_infliximab_ solution (6.0 mg in 3.3 mL). The endotoxin level of the PEG reagent was determined to be less than 0.005 EU/mL. The solution was incubated at ambient temperature for approximately 3 h without shaking while maintaining sterility. FpF_infliximab_ was purified using a single step HiTrap Macrocap SP cation exchange column (IEC-Macrocap SP, 5.0 mL). The IEC-Macrocap SP column was first washed with NaOH (1 M) and then ET free water before sample loading. After sample loading, the column was first eluted with 100% acetate buffer A for 10 min followed by a 30 min linear gradient using 100% acetate buffer B containing 1.0 M NaCl with a flow-rate of 1.5 mL/min. Fractions (1.5 mL) were analysed by SDS-PAGE and the FpF_infliximab_ fractions pooled. Using viva-spin (Turbo 15, MW cut off 30 kDa), the FpF_infliximab_ was concentrated to 4.0 mg/mL in a total volume of 0.2 mL. The concentration of the purified FpF_infliximab_ was calculated by micro BCA assay using infliximab as standard. The endotoxin level of the concentrated FpFinfliximab was determined to be less than 0.005 EU/mL using the EndoZyme assay according to manufacturer’s instructions (Hyglos, Munich, Germany).

### Surface Plasmon Resonance (SPR) Binding studies

Sensor chip NTA (nitrilotriacetic acid) is made of carboxymethylated dextran which is covalently immobilised with NTA. The surface of sensor chip NTA was first activated with nickel (Ni^+2^) solution to create the nickel chelated NTA group which allowed capture of histidine-tagged TNFα. Samples (FpF_infliximab_ or infliximab) were passed over the surface and the binding kinetics were determined. Between runs, the NTA surface was regenerated with EDTA solution to refresh the sensor chip for a new analysis cycle, starting with fresh NiCl_2_ solution and histidine-tag TNFα. Conditions to run the kinetic study were as follows; flow rate of 30 μL/min, 200 second contact time for NiCl_2_ solution (0.4 mL), 250 second contact time for human histidine-tag TNFα (5 μg, 1.0 mL), 150 second contact time for series of infliximab solutions (0.2 mg/mL–0.0625 mg/mL), and FpF_Infliximab_ solutions (0.2 mg/mL–0.0625 mg/mL). The NTA chip was regenerated using EDTA solution (350 mM, 1.0 mL) for 60 seconds.

### Experimental Autoimmune Uveoretinitis (EAU) Induction and therapeutic intervention

Female B10.RIII mice were immunized subcutaneously in one flank with 50 μg RBP-3_161–180_ in phosphate buffered saline (PBS) emulsified with Complete Freund’s Adjuvant (CFA) supplemented with 1.5 mg/ml *Mycobacterium tuberculosis* complete H37 Ra (BD Biosciences, Oxford, UK) (1:1 vol/vol). The mice also received 1 μg *Bordetella pertussis* toxin (Tocris, Bristol, UK) intraperitoneally (i.p.).

For local administration of infliximab or FpF_infliximab_, intravitreal injections were performed on day 10 post-immunization. In brief, the eye was proptosed and held in position with a pair of forceps, while 15 μg of infliximab or FpF_infliximab_ diluted in 2 μl PBS was injected using a 33-gauge hypodermic needle (Esslab, Essex, UK). The injection site was treated with chloramphenicol and globe reposited.

### EAU Clinical Assessment

Using a method adapted from Paques *et al*.^54^ an endoscope with a 5 cm long teleotoscope of 3 mm outer diameter (1218AA; Karl Storz, Tuttlingen, Germany) was connected to a Nikon D80 digital camera with a 10-million pixel charge-coupled device image sensor and Nikkor AF 85/F1.8 D objective (Nikon, Tokyo, Japan), with an additional +4.00 dioptre magnifying lens. Through pupils dilated with topical tropicamide 1% and phenylepherine 2.5% (Minims, from Chauvin Pharmaceuticals, UK), and topical oxybropucaine 0.4% (Minims) and Viscotears (Novartis Pharmaceuticals, UK) for corneal anesthesia, images were obtained by direct corneal contact with the endoscope. Images were processed using Adobe Photoshop (Adobe Corporation, Mountain View, CA). Using an adapted clinical grading system, fundal images were scored according to inflammatory changes to the optic disc and retinal vessels in addition to retinal lesions and structural damage^24^. All scores were added together to calculate a final disease score.

### Isolation and flow cytometric assessment of retinal infiltrate

Each eye was dissected in 100 μl ice-cold HBSS with aqueous, vitreous and retina extracted using a limbal incision, lens extraction and transfer into a 1.5 mL tube. The tissue was mechanically dissociated by rapping the tube across a standard rack ten times before transfer into a 96-well 60 μm cell strainer plate (Merck Millipore, UK). This was centrifuged at 1200 rpm for 5 minutes, the retinal supernatants was aspirated and the remaining cell pellet transferred into a 96-well V-bottom plate for immuno-staining.

Cells were incubated with 24G2 cell supernatant for 10 minutes at 4 °C before incubation with fluorochrome-conjugated monoclonal antibodies against cell surface markers including, CD4 [RM4-5], CD8 [53–6.7], CD11b [M1/70], Ly6G [1A8] and CD45 [30-F11] at 4 °C for 20 minutes. Cells were resuspended in 7AAD, and dead cells excluded from analysis by gating on 7AAD negative cells. Cell suspensions were acquired using a 3-laser BD^TM^ LSR-II flow cytometer (BD Cytometry Systems, Oxford, UK). Analysis was performed using FlowJo software (Treestar, San Carlos, California). Cell numbers were calculated by reference to a known cell–standard, as previously reported^55^. Briefly, splenocytes at a range of known cell concentrations were acquired using a fixed and stable flow rate for 1 minute. Based on total cell number acquired during this time, a standard curve was generated and used to interpolate cell concentrations of ocular infiltrating cells acquired at the same flow rate and time.

### Cell culture and intracellular uptake assay

A spontaneously transformed mouse retinal pigment epithelium (RPE) cell line B6-RPE07^31^ was cultured in DMEM medium supplemented with 10% heat-inactivated fetal calf serum, 2% L-glutamine, 1 mM sodium pyruvate, 60 μM 2-mercaptoethanol, 100 U/ml penicillin and 100 μg/ml streptomycin (complete medium) at 37 °C in an atmosphere of 5% CO_2_. RPE cells were passaged with a split ratio of 1:5 using 0.05% trypsin-EDTA (Life Technologies, Paisley, UK), and allowed to recover for 2 days in complete medium prior to experiments.

Confluent monolayers of B6RPE-07 cells in 24-well plates were treated once with 250 μg infliximab or FpF_infliximab_ for 4 or 24 hrs. Following treatment, B6RPE-07 cells were washed twice with ice-cold PBS. Cells were lysed directly from the plate using 200 μl CelLytic M solution supplemented with protease inhibitor cocktail (both from Sigma), and incubated on ice with gentle shaking for 10 minutes. Finally, these whole cell lysates were centrifuged for 10 minutes, at 4 °C at 13,000 rpm and the supernatant lysate collected and stored at −80 °C. To ensure equal loadings, a bicinchoninic acid (BCA) protein assay was performed prior to SDS-PAGE, and samples prepared with 5× Laemilli buffer. For Western blot, 10 μg of cell lysate loaded per well and separated on a 8–16% SDS-PAGE gel (Biorad), and proteins transferred to a nitrocellulose membrane. Following blocking in 5% milk/TBS/Tween-20, the membrane was subjected to analysis using the anti-human IgG (Fab specific)-peroxidase antibody (1:1000) (A0293; Sigma) and β-actin antibody (Cell Signaling) followed by a HRP conjugated polyclonal anti-mouse IgG (1:2000) before visualization using the chemiluminescent method (GE Healthcare Life Sciences).

Total RNA from B6RPE-07 cells, and *ex-vivo* mouse retina and corneal tissue was isolated using TRIzol reagent (Life Technologies), treated with RQ1 RNase-free DNase before cDNA synthesis using the ImProm-II^TM^ Reverse Transcription System (Promega, Southampton, UK). cDNA was amplified using the Power SYBR^®^ Green PCR Master Mix Reagent (Life Technologies) on a StepOne™ Applied Biosystems Real-Time PCR System. Mouse primer sequences used were: *Fcgrt* F: AGCTCAAGTTCCGATTCCTG; R: GATCTGGCTGATGAATC^29^; *Gapdh* F: TTCACCACCATGGAGAAGGC; R: GGCATGGACTGTGGTCATGA.

### Statistical analyses

Data was analyzed with unpaired Student’s t-test (GraphPad Prism software, San Diego, CA). Data are generated as mean ± SEM and representative of at least 2 independent experiments. Values were considered statistically significant at *p < 0.05, **p < 0.005, ***p < 0.0005.

## Additional Information

**How to cite this article**: Khalili, H. *et al*. An anti-TNF-α antibody mimetic to treat ocular inflammation. *Sci. Rep.* **6**, 36905; doi: 10.1038/srep36905 (2016).

**Publisher's note**: Springer Nature remains neutral with regard to jurisdictional claims in published maps and institutional affiliations.

## Supplementary Material

Supplementary Information
