# Supplementary material for: An anti-TNF-α antibody mimetic to treat ocular inflammation
Source: Sci Rep. 2016 Nov 22;6:36905. doi: 10.1038/srep36905 (PMC5118814; doi:10.1038/srep36905)
Supplement: Supplementary Information [file srep36905-s1.pdf]

Supplementary Figures:

An anti-TNF-α antibody mimetic to treat ocular inflammation

Hanieh Khalili[2,3†], Richard W Lee[1,3], Peng T Khaw[3], Steve Brocchini[2,3*], Andrew D Dick[1,3*] and David A Copland[1,3†]

Figure S1

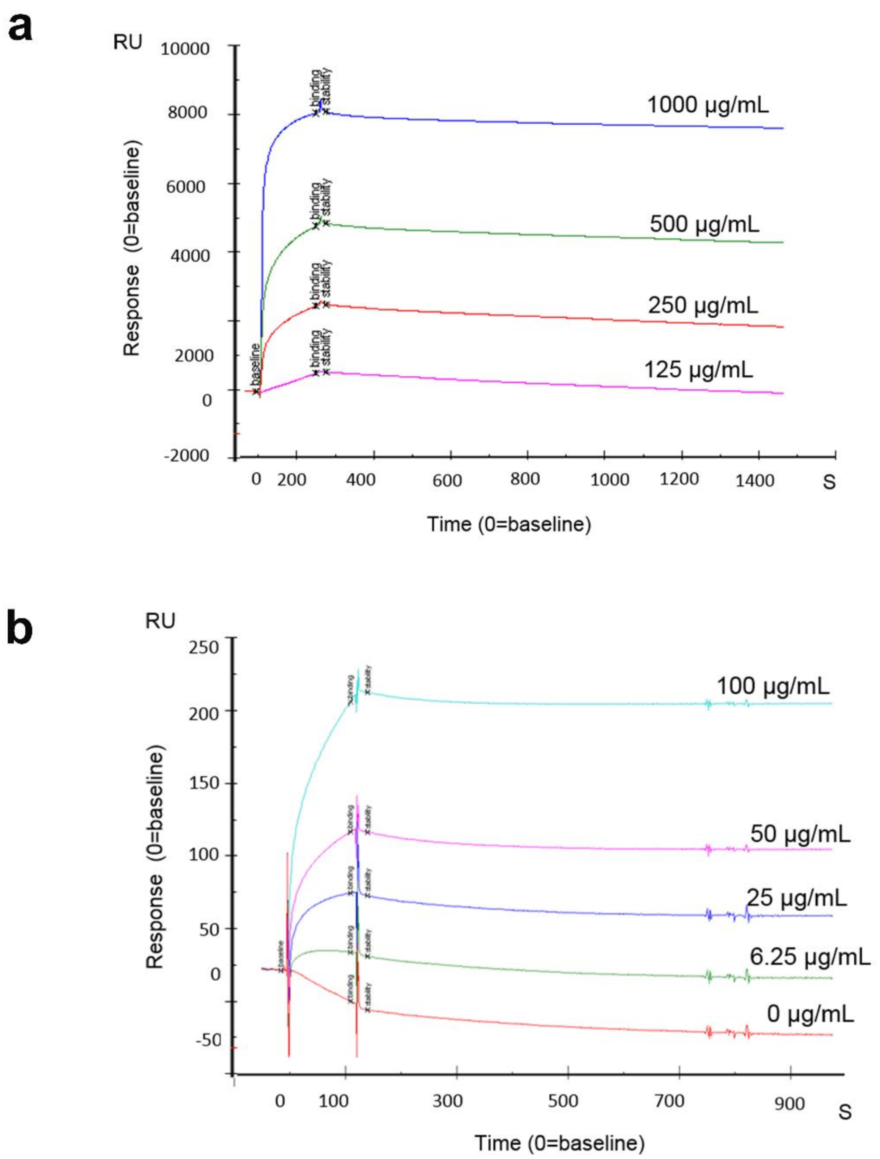

Figure S1: SPR binding assessment of infliximab and FpF$_{infliximab}$ to recombinant human TNF-α

Graphs detailing the Surface Plasmon Resonance (SPR) binding sensograms, confirming that infliximab **(A)** and FpF$_{infliximab}$ **(B)** can both bind to human TNF-α in a concentration-dependent manner. The NTA chip was functionalized with Ni solution first and then his-tag TNF-α (5μg/mL) solution prior to loading an anti-TNF-α molecules (infliximab and FpF$_{infliximab}$).

Figure S2

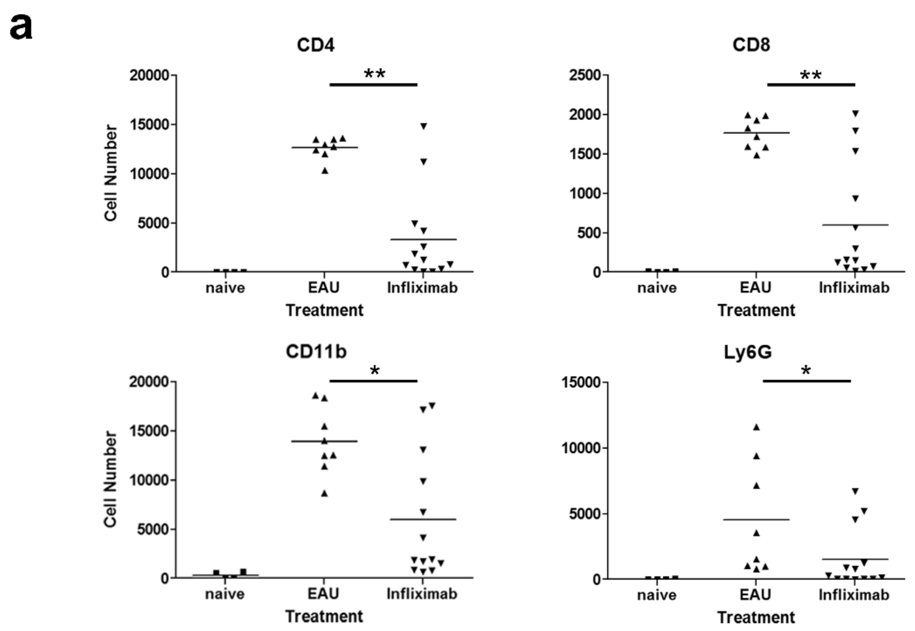

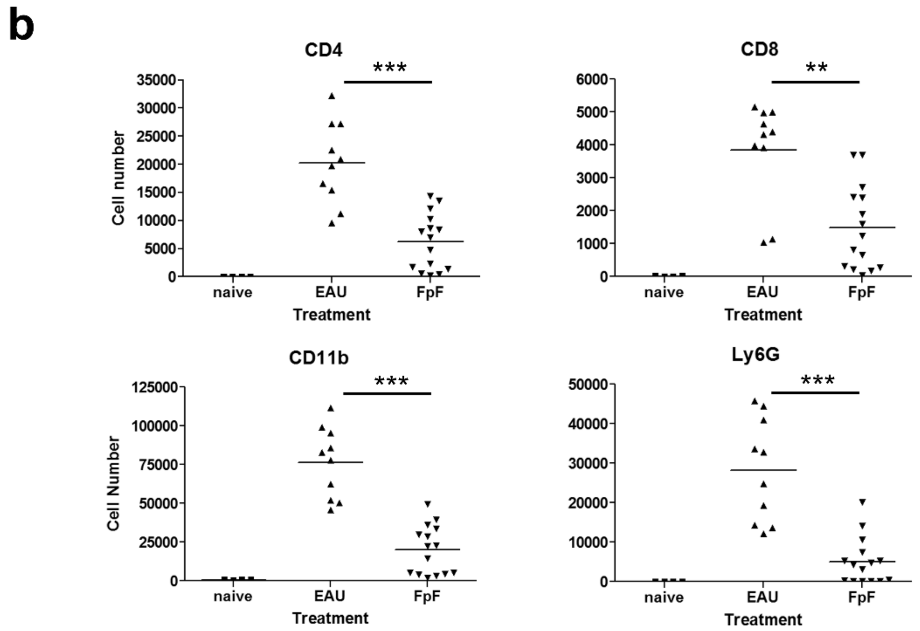

Figure S2: Analysis of retinal infiltrate following infliximab and FpF$_{infliximab}$ treatment

Groups of immunized mice received intravitreal injection of 15μg infliximab or FpF$_{infliximab}$ or vehicle control (EAU) on day 10. Eyes were enucleated on day 14, and retinal infiltrate characterized and quantified. Graphs detailing the specific CD45$^+$ subsets of retinal infiltrate (CD4$^+$, CD8$^+$, CD11b$^+$ and Ly6G$^+$). *P<0.05, **P<0.005, ***P<0.0005; Data presented as means ± SEM, and representative of two independent experiments.

Figure S3

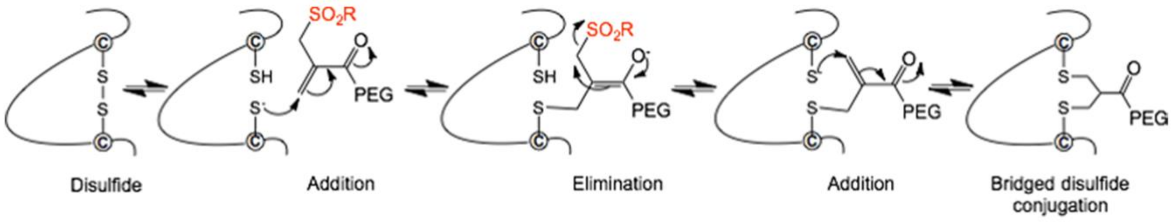

Figure S3: Bis-alkylation mechanism to generate three-carbon bridge site-specific conjugation between Fab and PEG scaffold.

The *mono*-sulfones **3** are latently crossed functionalized reagents capable of sequential and interactive addition-elimination reactions capable of bis-alkylation. In the case of disulfides, first the cysteine thiols are liberated by reduction (e.g. TCEP or DTT) and then conjugation involves (i) a first thiol addition to the mono-sulfone reagent **3**, (ii) sulphinic acid elimination to generate a second double bond, and (iii) a second thiol addition.

Figure S4

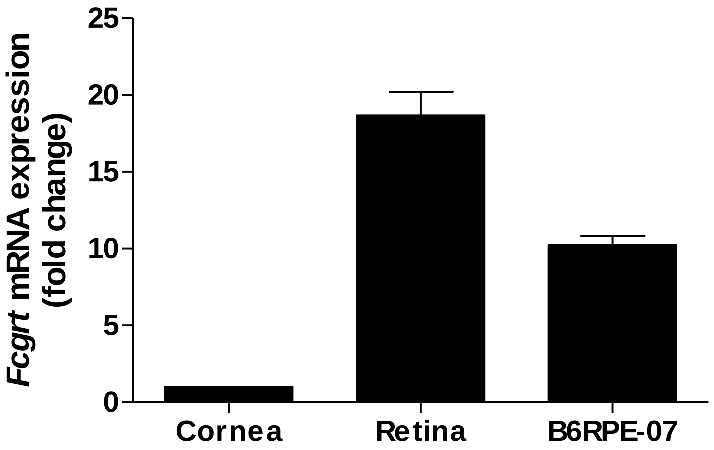

Figure S4: B6RPE-07 cell line expression Fcgrt mRNA

To confirm expression of the neonatal Fc receptor in the B6RPE-07 cell line, *Fcgrt* mRNA levels from 3 separate samples of cultured cells, as well as ex vivo retina and cornea tissues were determined by RT-qPCR. The ex-vivo mouse tissues were controls for *Fcgrt* expression, with retina (postive) and cornea (negative). Values were normalized to *Gapdh*, and the relative expression (fold change to negative control cornea) calculated.
